# Impact of Early Postnatal Androgen Exposure on Voice Development

**DOI:** 10.1371/journal.pone.0050242

**Published:** 2012-12-19

**Authors:** Leila Grisa, Maria L. Leonel, Maria I. R. Gonçalves, Francisco Pletsch, Elis R. Sade, Gislaine Custódio, Ivete P. S. Zagonel, Carlos A. Longui, Bonald C. Figueiredo

**Affiliations:** 1 Instituto de Pesquisa Pelé Pequeno Príncipe, Curitiba, Paraná, Brazil; 2 Hospital Pequeno Príncipe, Curitiba, Paraná, Brazil; 3 Faculdades Pequeno Príncipe, Curitiba, Paraná, Brazil; 4 Instituto da Voz de Maringá, Maringá, Paraná, Brazil; 5 Departamento de Fonoaudiologia da Universidade Federal de São Paulo, São Paulo, São Paulo, Brazil; 6 Clínica da Voz, Curitiba, Paraná, Brazil; 7 Irmandade da Santa Casa de Misericórdia de São Paulo, São Paulo, São Paulo, Brazil; 8 Departmento de Saúde Comunitária, Universidade Federal do Paraná, Curitiba, Paraná, Brazil; Ecole Normale Supérieure de Lyon, France

## Abstract

**Background:**

The impact of early postnatal androgen exposure on female laryngeal tissue may depend on certain characteristics of this exposure. We assessed the impact of the dose, duration, and timing of early androgen exposure on the vocal development of female subjects who had been treated for adrenocortical tumor (ACT) in childhood.

**Methods:**

The long-term effects of androgen exposure on the fundamental vocal frequency (F0), vocal pitch, and final height and the presence of virilizing signs were examined in 9 adult (age, 18.4 to 33.5 years) and 10 adolescent (13.6 to 17.8 years) female ACT patients. We also compared the current values with values obtained 0.9 years to 7.4 years after these subjects had undergone ACT surgery, a period during which they had shown normal androgen levels.

**Results:**

Of the 19 subjects, 17 (89%) had been diagnosed with ACT before 4 years of age, 1 (5%) at 8.16 years, and 1 (5%) at 10.75 years. Androgen exposure (2 to 30 months) was sufficiently strong to cause pubic hair growth in all subjects and clitoromegaly in 74% (14/19) of the subjects, but did not reduce their height from the target value. Although androgen exposure induced a remarkable reduction in F0 (132 Hz) and moderate pitch virilization in 1 subject and partial F0 virilization, resulting in F0 of 165 and 169 Hz, in 2 subjects, the majority had normal F0 ranging from 189 to 245 Hz.

**Conclusions:**

Female laryngeal tissue is less sensitive to androgen exposure between birth and adrenarche than during other periods. Differential larynx sensitivity to androgen exposure in childhood and F0 irreversibility in adulthood are age-, concentration-, duration-, and timing-dependent events that may also be affected by exposure to inhibitory or stimulatory hormones. Further studies are required to better characterize each of these factors.

## Introduction

Clinicians who are not voice specialists frequently examine vocal pitch as a proxy of perceptual vocal characteristics [1]. A more useful approach is to measure fundamental voice frequency (F0) in terms of cycles/sec (Hz), which provides a more precise estimation of the factors that cause linear changes in vocal characteristics. The female larynx is very sensitive to fluctuations in androgen levels, which decrease F0 to a level 1/3^rd^ lower than that of a child during puberty [2]–[4]. After puberty, the F0 of nonsmoking females reaches a plateau until the fifth decade, when it undergoes a slight decrease, most likely due to an increased testosterone-to-estrogen ratio after menopause [2], [5].

The mean F0 of the mature voice of women in southern Brazil, the majority of whom are of European descent, is 211 Hz [4]. In women undergoing androgen therapy, reduction in F0 or vocal pitch has been shown to be a function of androgen and testosterone levels [6]. Indeed, research has found that only 1 injection of testosterone may cause irreversible vocal changes in women [7], with 1 study of 38 women with CAH aged 18 to 63 years showing that these women had a significantly lower F0 than normal controls [8].

Although the greatest F0 reduction in men occurs during puberty, male F0 may still continue to decrease until the fourth decade, and may subsequently increase during the sixth decade [2], [9], [10]. Modifications in laryngeal size in adult men may also be caused by congenital growth hormone deficiency (GHD), which leads to development of an F0 of between 174 and 266 Hz, a range typical of normal adult women. The timing of GHD is a determining factor in pitch alteration, with research showing that patients who experience adult-onset GHD between 31 and 40 years of age have a normal male pitch and an F0 varying from 117 to 154 Hz [11].

The incidence of ACT in children younger than 15 years is at least 15 times higher in southern Brazil than that in the United States or Europe [12]–[15]; this trend is particularly prominent among girls younger than 4 years of age [15]–[17]. One major issue in this regard is the controversy surrounding the conditions under which virilization is irreversible in patients with pathological conditions. Therefore, this study aimed to identify the impact of variations in the intensity, duration, and timing of early postnatal androgen exposure on the final height, F0, and vocal pitch of female subjects. These female subjects had presented with ACT early in life and were subsequently reevaluated. As such, this is the first study to present data on the long-term impact of limited exposure to high androgen levels at a very young age among female subjects.

**Table 1:** Timing and characteristics of virilization at ACT diagnosis and time of study.

Subject	Age (years)	Age at ACT diagnosis (years)	Length of virilizationbefore diagnosis (months)	Time betweenACT andstudy (years)	Tanner stageat ACT diagnosis	Clitoral enlargement?	Age at menarche (years)	Height (FS)	FS Z-score	Target height (TH)	TH Z-score	Initial F0 0.9 to 7.4y after ACT Dx/expected F0(Hz)	Present F0 11 to 15 (Hz)	Vocal pitch
1(1)	21.1	10.7	8	10.41	P5	Yes	13	162.5	−0.13	156.1	−1.12	240/232	205	F
2	20.6	1.83	4	19.13	P3	Yes	12	165	0.26	167	0.57	233/232	216	F
3	13.6	1.16	2	12.42	P2	Yes	11	164.5	0.79	161	0.27	284/283	210	F
4(1)	17.8	2.75	4	14.81	P3	Yes	9	160.2	−0.45	155	−1.25	276/272	205	F
5	16.0	1.41	5	14.58	P3	Yes	10	160	−0.39	162	−0.08	304/275	245	F
6(1)	20.6	1.83	15	18.75	P3	Yes	13	158	−0.82	158.5	−0.75	214/232	199	F
7	18.4	2.16	6	16.25	P2	Yes	11	168.6	0.84	158	−0.80	203/247	165	M+
8	17.3	0.91	3	16.41	P3	No	12	164.1	0.17	165.3	0.36	228/275	238	F
9	15.1	1.5	3	13.51	P3	Yes	12	161.4	−0.08	163.2	0.20	221/275	199	F
10	13.9	2.16	10	11.67	P4	Yes	11	155	−0.77	153.5	−1.00	111/283	169	M+
11	16.1	2.25	17	13.81	P4	Yes	11	150.9	−1.81	157.5	−0.78	219/280	225	F
12	15.9	1.0	11	14.8	P4	Yes	14	164	0.23	158	−0.69	NA	217	F
13	21.9	3.16	6	18.74	?	?	11	166.4	0.47	153.5	−1.51	NA	185	F
14	20.4	8.16	30	12.25	?	Yes	9	152	−1.74	160.5	−0.44	NA	132	M++
15	33.5	1.50	8	32.25	?	?	12	158	−0.82	156.5	−1.05	NA	217	F
16	15.1	2.50	13	12.66	P2	Yes	14	165	0.48	165	0.48	NA	199	F
17	14.8	2.25	12	12.41	P2	?	13.5	158.5	−0.48	160.2	−0.22	NA	211	F
18	31.1	2.66	6	28.50	?	Yes	12	170.8	1.16	162.4	−0.14	NA	189	M+
19	25.2	3.91	16	21.34	P2	No	13	168	0.72	165	0.26	NA	216	F
Mean	19.58	2.93	9.42	16.56			11.76	161.73	−0.12	159.91	−0.40	−	202	
SD	5.61	2.51	6.80	5.70			1.48	5.44	0.83	4.09	0.65	−	26	

(1) Cushing’s syndrome. F: female pitch; M+: mild virilization; M++: moderate virilization; NA: not available. Initial F0 was obtained several weeks and 4 years after ACT diagnosis, as reported by Leonel in her thesis in 2003 [4].

**Table 2:** F0 and vocal pitch of control group.

Subject	Age (years)	F0	Vocal pitch
1	21.5	215.3	F
2	23.9	202.4	F
3	17.5	219.7	F
4	26.5	244.5	F
5	26.1	231.2	F
6	21.2	207.3	F
Mean	22.8	220.1	
SD	3.3	15.6	

## Materials and Methods

### Subjects

Before the study initiation, approval for this study was obtained by the Ethics Committee of Pequeno Príncipe Hospital and from each subject or, if the subject was a minor, from a parent, by requesting the subject or her parent to read and sign the consent form. Thirty female patients who had completed more than 11.4 years of ACT treatment at the main reference hospitals of Curitiba, the capital of Paraná State, were invited to participate. Of the 22 volunteers who had ACT and agreed to participate, 19 met the inclusion criteria of (1) having experienced virilizing symptoms related to a previously treated adrenocortical tumor in the pre-pubertal stage, with 16 having been treated for adrenocortical carcinoma and 3 for adenoma; (2) being at least 13 years of age, and (3) having experienced menarche at least 2 years prior to study initiation. All 19 also met none of the exclusion criteria of (1) being a smoker, (2) having undergone vocal treatment, (3) currently experiencing edema of the vocal folds for other reasons, or (4) having experienced prolonged androgen exposure due to multiple ACT recurrence. Of the 19 subjects, whose age ranged from 13.6 to 33.5 years, 9 were adult females ranging from 18.4 to 33.5 years of age and 10 were adolescents ranging from 13.6 to 17.8 years of age. Comparison of actual height and predicted target height (TH) and vocal assessment were conducted for all subjects.

**Figure 1:**
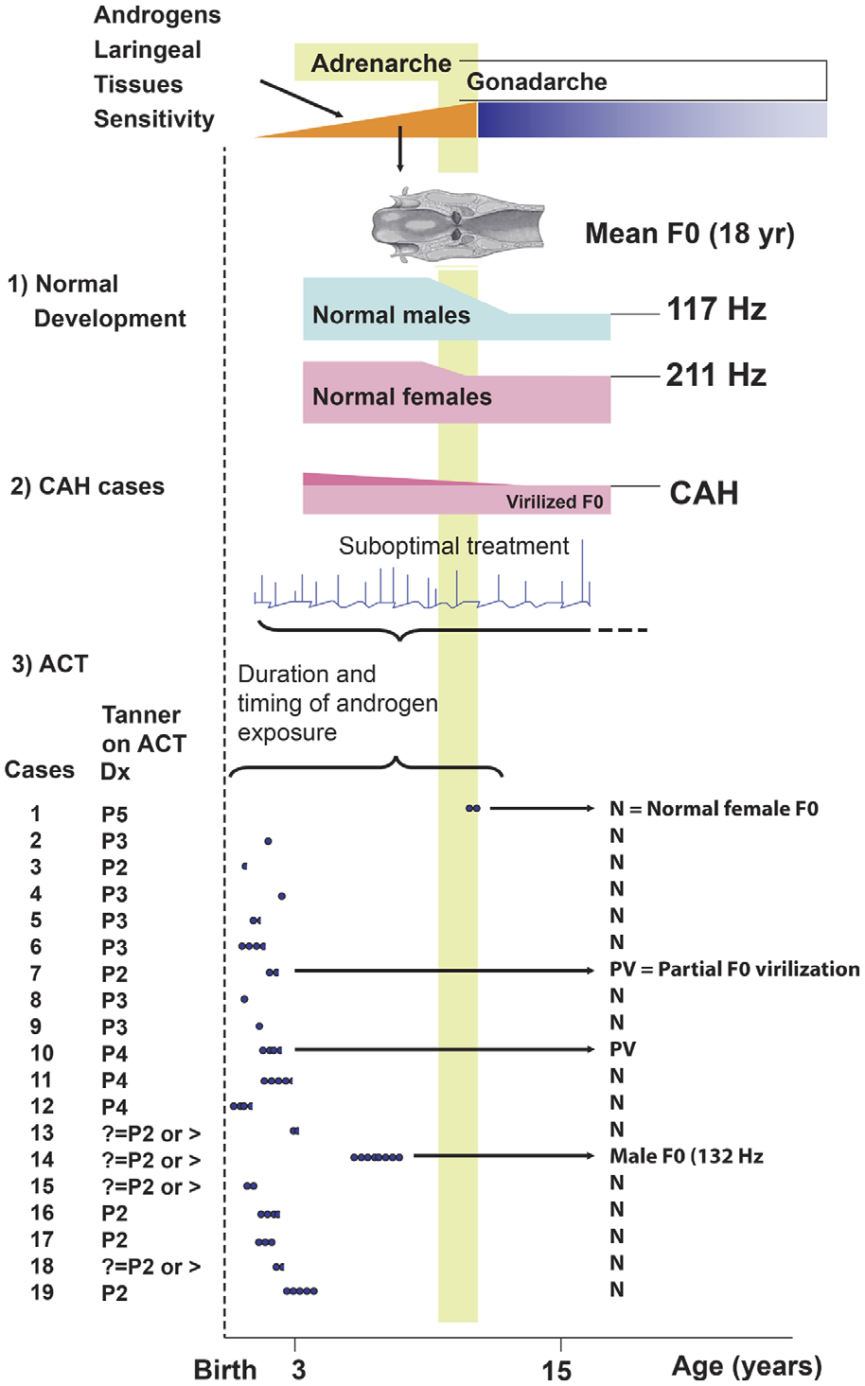
A flow chart summarizing the results of this and other studies investigating the postnatal periods of lowest and greatest larynx sensitivity to androgen exposure and the mechanism of vocal development in response to this exposure. Pink curves correspond to female F0 spectra and blue curves to male F0 spectra. (1) Normal development of F0 differentiation in males and females. Adrenarche and activation of the hypothalamus–pituitary–gonadal axis (gonadarche) are the main events leading to attainment of adult vocal patterns. At puberty, the larynx, vocal folds, and vocal tract (e.g., the resonance tube up to the oral cavity) acquire increased mass, leading to variable degrees of F0 reduction in men at adrenarche and gonadarche and to partial reduction in women at adrenarche. F0 decreases and then stabilizes between the ages of 14 and 18 years. Mean F0, which is shown for normal male and female Brazilian subjects [4], slightly decreases with age, as was shown in a study of normal Swedish women [2] and in other studies (Hollien and Shipp, 1972; Stoicheff, 1981; Hollien et al., 1994), due to steroids and other factors. (2) A hypothesis based on the study results postulating that androgen exposure during pre- and post-pubertal stages in CAH females causes fluctuations in androgen levels and permanent virilization of F0. (3) A good marker identified for the accumulated androgen effect (represented by Tanner stage) is shown for all ACT cases. Each full dot represents 4 months of androgen exposure. Androgen exposure during the pre-pubertal stage leading to decreased F0 was found in only 3 ACT cases (3/19; 15.7%), leading to development of the hypothesis that laryngeal tissue has a low level of sensitivity to androgen exposure during the first 5 postnatal years in females.

Of the 19 subjects, 17 (89%) had been diagnosed with ACT before 4 years of age, 1 (5%) at 8.16 years, and 1 (5%) at 10.75 years. The majority (15/19, 78%) had participated in a molecular analysis of ACT patients in which they had tested positive for the germline R337H mutation in the *TP53* gene [12], [13], [18]–[20]. Three of the participants (16%) showed a combination of virilization and Cushing’s syndrome (CS) at the time of ACT diagnosis. The characteristics of external genitalia at early ages and the duration over which the patients showed clinical manifestations of androgen-induced virilizing syndrome before ACT diagnosis, which ranged from 2 to 30 months, were determined from medical records. A control group composed of 6 female siblings of the participants, whose age ranged from 17.5 to 26.5 years, was recruited to serve as representatives of the normal female population. In addition, the F0 data obtained from the normal female population in this region by one of the coauthors served as valuable control data (ML’s thesis, 2003, reference 4).

### Vocal Recordings

Vocal recordings were conducted in a speech pathology outpatient clinic room free of noise and echo. While seated with her feet on the ground, her posture upright, and a digital recorder (IMP Optimus Unidirectional Microphone 600Ω; Philippines) placed 10 cm from her mouth at a 90° angle from her chin, each participant was recorded continuously vocalizing the “e” vowel sound while counting from 1 to 20. In accordance with the observations of recording manufacturers and other authors, sustained vocalization of the “e” vowel sound was selected from among all possible vocalizations because it provides for the best possible capture [8], [21]–[24]. The software packages used for acoustic measurement were the Software Quality Voice Files for Vowels program and the Analysis of Voice at the Numbers program (VoxMetria® version 4.6). Perceptual evaluation of pitch was conducted by the same clinic and vocal recordings were reviewed by evaluators blinded to the study protocol at a second voice laboratory located at the Federal University of São Paulo (UNIFESP).

The F0 values were compared to the results of previous analyses of recordings of 11 subjects, which had been made from 0.9 years to 7.4 years after these subjects had undergone ACT surgery; during this period, these subjects had presented with normal androgen levels [4]. Although 1 participant had experienced local relapse several months after removal of the primary ACT, she had experienced no metastasis and been successfully treated with chemotherapy. Qualitative evaluation of voice was also conducted by 1 physician and 2 speech pathologists previously trained to conduct vocal ratings, who independently evaluated vocal pitch by rating the tone as manifesting mild virilization (+), moderate virilization (++), or a typical male pitch (+++). Laryngoscopy and vocal analysis were performed simultaneously with F0 and pitch evaluation to monitor for potential laryngeal pathology.

### Statistical Analysis

Using reference population data from the National Center for Health Statistics (NCHS) [25], each participant’s height value was transformed to an actual height Z-score (the standard deviation score of the mean) and the value of the participant’s parent’s height was used to calculate her TH Z-score [26], [27]. Comparison of the subject’s actual Z-score and TH Z-score was performed using the paired Student’s t-test for dependent samples, with a final height Z-score up to 1.0 standard deviation above or below the target height Z-score considered an indicator of accordance with the genetic pattern in height [28].

## Results

### Physical Examination

None of the subjects had experienced any pathological condition other than ACT or taken any anabolic steroids. At ACT diagnosis, all the subjects (19/19) presented with pubic hair growth and the majority (14/19, 74%) presented with clitoromegaly. In addition, 3 subjects, patients 1, 4, and 6, presented with signs of CS, specifically with virilizing signs, since 8, 4, and 15 months before diagnosis, respectively, and these signs had been confirmed to be CS indicators by subsequent measurement of mildly or modestly elevated cortisol levels. The height of all the subjects fell within the 50^th^ and 75^th^ percentiles, corresponding to their familial TH, including that of the 1 participant (subject 14) who presented with the lowest F0 (132 Hz) and moderate pitch virilization.

### Vocal Characteristics

As previously described, 57% (11/19) of the participants had previously participated in an analysis of F0 values obtained from 0.9 years to 7.4 years after ACT surgery. At this early age, 54% (6/11) had presented with a normal F0 and 45% (5/11) had F0 significantly lower than the expected F0 for their chronological age. This reduction in F0 was found to be inversely correlated (p<0.05) with Tanner stage [4], which was used as a parameter of accumulated androgen effect until ACT diagnosis.

Table 1 also presents the initial F0 of the subjects for whom data were available. As can be observed, initial data were available for only 11 of the subjects because several patients did not survive, experienced multiple recurrences, or were not eligible for study inclusion. The reliability of the instrumental measure for F0 was found to be more accurate and quantitative than the subjective evaluation (pitch). Mild or moderate virilization of pitch was observed in the 4 subjects (subjects 7, 10, 14, and 18) who had presented with the lowest F0 (165, 169, 132, and 189 Hz, respectively), although subject 18 had presented with an F0 within the lower limit of normality for females. Investigation of the extent to which the marked reduction in F0 was irreversible, which was performed by measurement of F0 more than 11 years later, indicated that 3 subjects (subjects 8, 9, and 11) experienced complete recovery of F0 and 2 subjects (subjects 7 and 10) experienced only partial recovery, which accounted for the mild pitch virilization in these subjects (Table 1). Interestingly, the F0 of patient 10 had increased from 111 Hz to 169 Hz. One adult subject (subject 14) with an F0 typical of that of males (132 Hz) had been diagnosed with ACT at the age of 8.16 years and had experienced virilizing syndrome for 2.5 years before ACT diagnosis. However, as shown in Table 1, the observed reduction in F0 in children only 0.9 years to 7.4 years after ACT treatment is not a permanent effect leading to virilized F0 in mature subjects. Sixteen (16/19, 84%) subjects had been diagnosed with ACT before 4 years of age and had experienced virilizing syndrome for 2 to 17 months. One subject (1/19, 5%) who had undergone 8 months of androgen exposure before diagnosis at the age of 10.7 years was found to have a normal adult female F0 comparable to that of most cases in the present study (16/19), the control group (Table 2), or the control group previously described [4].

## Discussion

Increased production of sex hormones in normal female subjects during adrenarche and gonadarche induces growth of the larynx, causing a rapid decrease in vocal pitch. Despite knowledge of this phenomenon, little is known regarding the sensitivity of the female larynx to androgen exposure during the pre-pubertal stage. This knowledge gap is primarily due to the absence of F0 analysis not only in the rare cases of childhood ACT [15], [16], but also in the few cases of childhood virilization caused by topical exposure (usually accidental) to testosterone in the form of gels or patches [29]–[31]. To our knowledge, this study is the first long-term analysis of F0 in female patients exposed to high levels of androgens at a very young age. We examined the effect of the timing of this exposure, specifically whether androgen exposure causes significant virilization during the pre-pubertal period (ranging from few months after birth to adrenarche).

Due to the fact that survivors of multiple ACT recurrence were excluded from the study because of prolonged androgen exposure, as were smokers and patients who presented with signs of inflammatory throat lesions, as well as the existence of a low ACT survival rate [16] and follow-up losses, only 19 ACT patients were eligible for study participation. Nevertheless, the foremost research goal–determination of the impact of androgen exposure leading to the development of remarkable pre-pubertal virilizing signs on long-term (adult or adolescent) female F0–was achieved. All the subjects (19/19) demonstrated a good understanding of ACT signs and symptoms, especially due to their participation in previous studies [12], [13], [32]. The variables of dose, timing, and duration of androgen exposure were all found to be determinants of virilization in the subjects, of whom 18 had experienced virilization during the pre-pubertal stage and 1 during puberty. Virilization was remarkable because of different degrees of pubic hair growth (19/19, 100%) and its association with clitoral enlargement in the majority of the subjects (14/19, 74%). All the subjects reported that their medical history had been uneventful after ACT treatment, and most reported that their clitoris had partially returned to normal size, although this parameter was not reevaluated in the most recent physical exam.

Although all the subjects had experienced a relatively long period (2 to 30 months) of androgen exposure in childhood, the exposure had not been sufficiently strong to cause loss of TH in any of the subjects. Furthermore, the observed decrease in F0 in 45% (5/11) of the subjects within 0.9 to 7.4 years after tumor resection, i.e., after normalization of androgen levels [4], had subsequently recovered to normal values in 3 cases, and only 18% (2/11; subjects 7 and 10) presented with partial virilization of F0 (165 and 169 Hz, respectively) and mild virilization of pitch. Interestingly, 1 of these 2 cases (1/11) had presented with a typical adult male F0 in childhood, suggesting that F0 may also be partially recovered (from 111 Hz to 169 Hz) in the absence of any treatment. Observation of mild or moderate virilization of pitch observed in 4 participants was consistent with the lowest detected F0, which ranged from 189 to 132 Hz. However, as expected, the method used to measure F0 was more reliable (and quantitative) than that used to evaluate pitch.

Although F0 has previously been investigated in female subjects with CAH [8], these subjects may have been periodically exposed to increased levels of androgens due to suboptimal treatment with glucocorticoids [33], making it difficult to identify the timing and duration of androgenic effects and making these subjects less than ideal models. Figure 1 describes the timing and degrees of androgen exposure (Tanner stage) as a reflection of the intensity of the accumulated androgen effect. The determinants of laryngeal tissue sensitivity to androgens and extent of differentiation shown in Figure 1 were defined on the basis of the results of this study and previous studies [1], [3], [4], [8], [9], [10]. Lack of analysis regarding early androgen exposure, particularly during the initial development of ACT symptoms, combined with differences found among the types of androgens examined has prevented systematic comparison of hormone levels. Despite this challenge, the findings of this study reveal the differences between the impact of ACT and CAH, specifically that androgen exposure during early childhood is less likely to result in an irreversibly virilized F0, and any significant reductions in F0 at an early age are likely to be partially reversed by increases to a normal F0 between childhood and adulthood.

Previous authors have proposed the existence of an androgenic effect that includes the development of increased mass in laryngeal tissue. Current knowledge of the mechanism by which steroids induce sexual differentiation of vocal production at the brain level is limited to animal models, particularly songbirds [34] and female African clawed frogs (*Xenopus laevis*) [35], [36]. The female laryngeal muscle differs from the male laryngeal muscle [37], as most female laryngeal neuromuscular synapses are stronger than the corresponding male synapses [38]–[40]. At the CNS level, these differences are responsible for generating sexually dimorphic vocalizations, which are recorded in motor neurons [35].

The study data shown in Figure 1 support the hypothesis that a period of low sensitivity in laryngeal tissue exists between birth and adrenarche. The experience of subject 14, who had developed ACT at the onset of or before adrenarche, with her parents first reporting virilizing signs when she was less than 6 years old, provides particular evidence of this phenomenon. Moreover, subject 14 had also been exposed to particularly high levels of androgens for a prolonged period lasting until close to the onset of adrenarche, which, being a period of tissue maturation in the larynx, had increased the probability that she would experience vocal virilization.

As shown in Table 1, 3 subjects presented with CS, as confirmed by measurement of mild to modest elevations in cortisol levels. However, it is unclear whether the elevation in serum cortisol concentration impacts the effects of androgen on laryngeal tissue. Therefore, further studies are necessary to evaluate the effects of the concentration, duration, and timing of cortisol exposure on the F0 of children with CS.

In conclusion, the findings of this study support the hypothesis that female laryngeal tissue is less sensitive to androgen exposure between birth and adrenarche. Particularly compelling was the finding of F0 virilization in only 1 subject, who had experienced androgen exposure immediately before the period at which adrenarche typically begins and for a longer duration (30 months) than that in the other subjects. Although a subject who had experienced androgen exposure during adrenarche had subsequently developed a normal female F0, she had also experienced elevated cortisol levels during the same period (8 months before ACT diagnosis) based on the reported clinical signs. Thus, the possibility that cortisol production had exerted an inhibitory effect on androgen exposure cannot be ruled out in this case. Furthermore, it was not possible to define the exact contribution of ACT androgens, which most likely acted during the final stages of adrenarche. The findings also indicate that differential larynx sensitivity to androgens exposure in childhood and F0 irreversibility later in adulthood are age-, androgen concentration-, duration-, and timing-dependent events that may also be affected by exposure to inhibitory or stimulatory hormones, such as glucocorticoids. Further studies are now required to better characterize each of these factors.
